# Optimizing the detection of emerging infections using mobility-based spatial sampling

**DOI:** 10.21203/rs.3.rs-3597070/v1

**Published:** 2023-11-17

**Authors:** Die Zhang, Yong Ge, Jianghao Wang, Haiyan Liu, Wen-Bin Zhang, Xilin Wu, Gerard B. M. Heuvelink, Chaoyang Wu, Juan Yang, Nick W. Ruktanonchai, Sarchil H. Qader, Corrine W. Ruktanonchai, Eimear Cleary, Yongcheng Yao, Jian Liu, Chibuzor C. Nnanatu, Amy Wesolowski, Derek A.T. Cummings, Andrew J. Tatem, Shengjie Lai

**Affiliations:** a School of Geography and Environment, Jiangxi Normal University, Nanchang, China; b State Key Laboratory of Resources and Environmental Information System, Institute of Geographic Sciences & Natural Resources Research, Chinese Academy of Sciences, Beijing, China; c Key Laboratory of Poyang Lake Wetland and Watershed Research Ministry of Education, Jiangxi Normal University, Nanchang, China; d University of Chinese Academy of Sciences, Beijing, China; e Ocean Data Center, Southern Marine Science and Engineering Guangdong Laboratory (Zhuhai), Zhuhai, China; f WorldPop, School of Geography and Environmental Science, University of Southampton, Southampton, UK; g ISRIC - World Soil Information, Wageningen, the Netherlands; h Soil Geography and Landscape Group, Wageningen University, Wageningen, the Netherlands; i The Key Laboratory of Land Surface Pattern and Simulation, Institute of Geographical Sciences and Natural Resources Research, Chinese Academy of Sciences, Beijing, China; j School of Public Health, Fudan University, Key Laboratory of Public Health Safety, Ministry of Education, Shanghai, China; k Shanghai Institute of Infectious Disease and Biosecurity, Fudan University, Shanghai, China; l Population Health Sciences, Virginia Tech, Blacksburg, VA, USA; m Natural Resources Department, College of Agricultural Engineering Sciences, University of Sulaimani; Sulaimani 334, Kurdistan Region, Iraq; n School of Mathematics and Statistics, Zhengzhou Normal University, Zhengzhou, China;; o Department of Epidemiology, Johns Hopkins Bloomberg School of Public Health, Baltimore, MD, USA; p Department of Biology and Emerging Pathogens Institute, University of Florida, Gainesville, FL, USA; q Institute for Life Sciences, University of Southampton, Southampton, UK.

**Keywords:** human mobility, spatial sampling, testing, emerging infectious disease

## Abstract

**Background:**

Timely and precise detection of emerging infections is crucial for effective outbreak management and disease control. Human mobility significantly influences infection risks and transmission dynamics, and spatial sampling is a valuable tool for pinpointing potential infections in specific areas. This study explored spatial sampling methods, informed by various mobility patterns, to optimize the allocation of testing resources for detecting emerging infections.

**Methods:**

Mobility patterns, derived from clustering point-of-interest data and travel data, were integrated into four spatial sampling approaches to detect emerging infections at the community level. To evaluate the effectiveness of the proposed mobility-based spatial sampling, we conducted analyses using actual and simulated outbreaks under different scenarios of transmissibility, intervention timing, and population density in cities.

**Results:**

By leveraging inter-community movement data and initial case locations, the proposed case flow intensity (CFI) and case transmission intensity (CTI)-informed sampling approaches could considerably reduce the number of tests required for both actual and simulated outbreaks. Nonetheless, the prompt use of CFI and CTI within communities is imperative for effective detection, particularly for highly contagious infections in densely populated areas.

**Conclusions:**

The mobility-based spatial sampling approach can substantially improve the efficiency of community-level testing for detecting emerging infections. It achieves this by reducing the number of individuals screened while maintaining a high accuracy rate of infection identification. It represents a cost-effective solution to optimize the deployment of testing resources, when necessary, to contain emerging infectious diseases in diverse settings.

## Background

Over the last few decades, emerging infectious diseases (EIDs) have more frequently become epidemic or pandemics more regularly in this highly mobile, ever-connected world, including severe acute respiratory syndrome coronavirus (2003), H1N1 influenza (2009), Middle East respiratory syndrome (2012), Ebola virus disease in West Africa (2013–2016), Zika virus disease (2015), and the coronavirus disease in 2019 (COVID-19) caused by severe acute respiratory syndrome coronavirus 2 (SARS-CoV-2) and its variants [1]. Timely and accurate identification of infected individuals is crucial for the effective containment and management of EIDs [2]. However, identifying all infectious individuals among populations, especially for diseases caused by highly contagious pathogens, can present significant resource and cost challenges. The spread of infectious diseases is closely linked to variations in human activities, underscoring the value of mobility patterns in effectively testing and identifying potential cases in a cost-effective manner at community level.

Due to the substantial risk of asymptomatic transmission and the rapid dissemination of severe illnesses within populations, a proactive testing approach, such as mass testing, has demonstrated its importance in infection detection [3, 4]. Subsequent interventions, such as isolation and contact tracing, are then implemented to mitigate transmission both within and between communities. For instance, during the COVID-19 pandemic, countries utilized mass testing through polymerase chain reaction assays and distributed lateral flow test kits, facilitating timely detection and isolation of infections across various settings [5–7]. Efficiently optimizing citywide screenings across spatial and temporal dimensions is crucial to address challenges such as cost constraints, limited healthcare infrastructure, logistical complexities, and community intervention fatigue [8]. However, the strategic selection of target populations for testing in spatial domains often lacks comprehensive optimization [9–12]. For example, prioritizing testing resources for individuals residing in close proximity to known cases, compared to those in disease-free regions, aligns with the diverse transmission modes of EIDs. Spatial sampling, integrating the spatial structure of the target, offers superior sampling accuracy and efficiency compared to the widely used simple random sampling (SRS) approach [13]. Therefore, combining spatial sampling with disease transmission characteristics can provide valuable information on target populations at risk, enabling the optimization of the allocation and deployment of testing resources.

In outbreaks involving human-to-human transmission, infection risks and population-level spread are significantly influenced by individual movement and contacts [14–16]. Leveraging information on individuals’ movement and contact behavior enhances spatial sampling’s targeting precision towards locations with a heightened likelihood of infections. Increasingly, human mobility and Point-of-interests (POIs) data are leveraged in infectious disease responses and analyses, encompassing activities such as close contact tracing [17, 18], risk prediction for transmission [19, 20], assessment of behavioral and emotional shifts in populations [21, 22], and evaluation of non-pharmaceutical intervention impacts [23–26]. However, these aspects are seldom factored into the determination of locations and population groups for screening in current pandemic testing, especially at fine spatial scales [27–29]. Real-time or near real-time mobility data holds promise in tailoring precise, population-wide testing strategies [30, 31].

In this study, we devised a mobility-based spatial sampling framework aimed at detecting EIDs that propagate through community transmission. Leveraging hourly mobile phone signaling data and comprehensive POI data, we quantified individual movement patterns and contact intensity, enabling estimation of disease transmission within communities, represented as community-level infection risk. We compared and designed four sampling approaches—human contact intensity (HCI), human flow intensity (HFI), case flow intensity (CFI), and case transmission intensity (CTI)—each employing distinct data requirements and measurements of human mobility characteristics (see Materials and Methods). To evaluate the performance of these mobility-based sampling approaches, we used the data of COVID-19 outbreaks in Beijing and Guangzhou, China, alongside simulated outbreaks under varying scenarios of transmissibility, interventions, and population density. Our evaluation encompassed a comparison with outcomes from SRS, citywide screening, and the utilization of a Susceptible-Exposed-Infectious-Removed (SEIR) epidemiological model. Furthermore, we assessed how the optimized spatial sampling approaches enhance the implementation of multi-round testing across diverse geographic ranges and temporal frequencies. Our proposed approaches, CFI and CTI, stand as valuable references for more economical allocation of testing resources and early surveillance of intra-city transmission, facilitating the effective control of EIDs across diverse settings.

## Materials and Methods

### Data sources

To assess the effectiveness of the proposed mobility-based sampling strategies in real-world scenarios of emerging infections, we gathered data on mobility, POI, demographics, and epidemiology concerning importation-related COVID-19 outbreaks in two cities, Beijing and Guangzhou, during the period of 2020–2021. The cities were subdivided into township-level divisions, which we considered as our sample units, referred to as communities in our study.

In Beijing, the first case of the COVID-19 outbreak was identified on June 11, 2020, following 56 consecutive days without a new confirmed case since the initial wave in 2020 [32]. The Xinfadi market was identified as the source of the outbreak, leading to its closure on June 13. By July 5, 2020, a total of 368 cases were reported in 52 affected communities, comprising 15.7% of all 331 communities. In Guangzhou, the first case of the highly transmissible VOC Delta variant of SARS-Cov-2 was confirmed in Liwan District on May 21, 2021 [33]. As of June 18, 2021, 16 communities in Guangzhou, accounting for 9.5% of 168 communities, had been affected, resulting in a total of 152 confirmed and asymptomatic cases. In both outbreaks, mass testing was promptly conducted after community transmission was confirmed to identify more infections and contain the outbreak. Ultimately, over 10 million people in Beijing [34] and 16 million residents in Guangzhou [35] were screened.

We acquired 2020 population data at a 100-meter resolution from WorldPop (www.worldpop.org). This data was then aggregated to estimate the population in each community using zonal statistics. Details on affected communities and case numbers were sourced from press releases and daily epidemic notification reports by the Beijing and Guangzhou Municipal Health Commissions (Additional file 1: Table S1).

To understand population movements between communities, we utilized anonymized data on population movement flows aggregated from cellular signaling data by China Mobile, a major mobile carrier in China (Additional file 1: Text S1). As of December 2021, China Mobile had 957 million users, representing 68% of the national population [36]. We aggregated hourly data from two specific days to capture population movement patterns between communities in Beijing on June 11 and 12, 2020, and in Guangzhou on May 21 and 22, 2021, respectively. These dates were during the early stages of the COVID-19 outbreaks and represent typical daily and normal mobility patterns prior to the enforcement of major travel restrictions. It’s important to note that the population flow data presented in this study provides hourly and inter-community flows of the general population and does not allow for individual tracking.

Regarding POI data for 2020, we obtained it from AMap Services (https://ditu.amap.com), a prominent location-based service provider in China. There was a total of 1,285,920 POIs in Beijing and 1,314,796 POIs in Guangzhou, each with six core fields: POI name, multilevel categories, address, coordinate location (latitude and longitude), and district name (Additional file 1: Fig. S9).

### Spatial sampling framework incorporating mobility and POI data

**Fig. 1.**
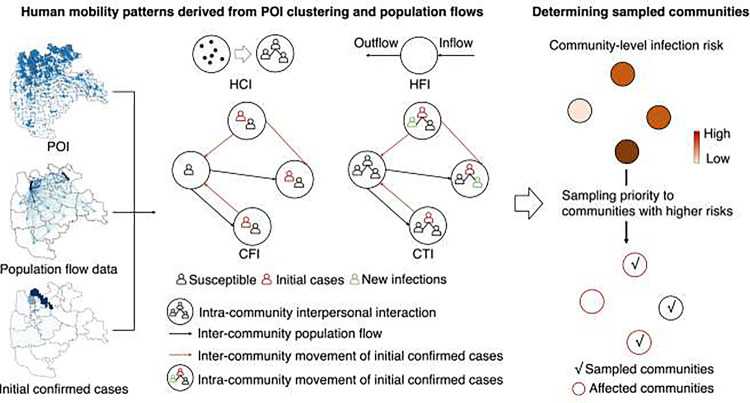
Framework of mobility-based spatial sampling approaches for detecting emerging infections at the community level. Utilizing data on Points of Interest (POIs), travel flows derived from mobile phone signaling, and the locations of initial confirmed cases within a city, four spatial sampling approaches were developed: Human Contact Intensity (HCI), Human Flow Intensity (HFI), Case Flow Intensity (CFI), and Case Transmission Intensity (CTI). The spatial sampling prioritizes communities based on infection risk ($\rho_{i}$), where communities with a higher $\rho_{i}$ are given higher sampling priorities.

We devised mobility-based spatial sampling methods utilizing mobile phone signaling and POI data to compute a community’s sampling priority and allocate testing resources at the community level. Fig. 1 provides an overview of the spatial sampling framework.

The sampling priority ($\rho_{i}$), representing the community-level infection risk due to COVID-19 transmission, was computed using data on mobile phone signaling geo-positions, POIs, and the location of initial confirmed cases. Different mobility scenarios derived from POI clustering and population flow data were incorporated into four spatial sampling approaches.

HCI (Human Contact Intensity) assessed the risk of transmission resulting from interpersonal contact within a community. It used a diversity index based on the number and category of POIs within a community to measure daily activity levels. POIs-based diversity indices have been widely used to depict the neighborhood vibrancy and human activity [37–39]. HFI (Human Flow Intensity) estimated spatial infection risk based on the movement of people entering and leaving a community, as represented by population inflow and outflow. Larger hourly population flows for a community indicated higher human contact risk and infection likelihood. HCI and HFI sampling focused on the daily contact and flow count within each community, respectively. These methods did not consider interactions between communities or utilize epidemiological data of the target disease.

CFI (Case Flow Intensity) leveraged a travel network to calculate $\rho_{i}$, using hourly counts of initial cases visiting a community by considering both the location of initial cases and their inter-community movements, derived from case and mobility data. This approach identified higher infection risk in communities that were visited by more cases. The travel network-based CTI (Community Transmission Intensity) utilized hourly counts of potential new infections, focusing on the risk introduced by intra-community contacts between cases and susceptible populations. Building upon CFI, CTI incorporated POI data to account for transmission events caused by cases in a community, identifying communities with a higher CTI where individuals were more likely to be infected.

Sample sizes were determined based on testing resource capacity, and communities with higher $\rho_{i}$ were given higher sampling priorities for ‘all residents’ screening under the specified sample size. Tests were conducted in the sampled communities, including affected communities where corresponding infections were detected.

In the context of mobility-based spatial sampling, we delineated four distinct approaches (HCI, HFI, CFI, and CTI) based on various human mobility characteristics to ascertain the infection risk ($\rho_{i}$). Additionally, we employed an epidemiological model to estimate the infection risk at the community level for comparative analysis. Each sampling approach was associated with a specific threshold and unit for $\rho_{i}$, facilitating a relative-level estimation of the extent of epidemic transmission within communities.

To better demonstrate individuals’ movement, all communities within a city were expressed as the set $S=\left\{s_{i},i=1,2,\ldots ,N\right\}$. At hour t, communities from which people go to the community $s_{l}$ were denoted as $M_{\to i}^{t}=\left\{s_{j}\in S,s_{j}\neq s_{i},0\leq \left|M_{\to i}^{t}\right|<N\right\}$, where $\left|M_{\to i}^{t}\right|$ was the number of elements in the set. Communities where people go from the community $s_{i}$ were denoted as $M_{i\to }^{t}=\left\{s_{k}\in S,s_{k}\neq s_{i},0\leq \right.\;\left.\left|M_{i}^{t}-\right|<N\right\}$. The number of visitors from $s_{j}$ to $s_{i}$ is $P_{ji}^{t}$, and the number of population inflow and outflow for the community $s_{i}$ was given by $P_{\to i}^{t}=\sum_{s_{j}\in M_{\to i}^{t}}P_{ji}^{t}$ and $P_{i\to }^{t}=\sum_{s_{k}\in M_{i\to }^{t}}P_{ik}^{t}$, respectively. Therefore, the number of people active in the community $s_{i}$ was computed as $P_{i}^{t}=P_{i}^{t-1}+P_{\to i}^{t}-P_{i\to }^{t}$, where the community-level population at hour t=0 (i.e., $P_{i}^{t-0}$) was the WorldPop-aggregated population.

#### Human contact intensity (HCI).

The infection risk considering interpersonal interaction within a community was depicted by a diversity index [40] based on the number and category of POIs, given by $\beta_{i}={\left(\sum_{c}\;{\left(m_{i,c}\right)}^{q}\right)}^{1/(1-q)}$, where $m_{i,c}$ is the number of POIs in the community $s_{i}$ for POI category c (i.e, secondary category in the study), and q is the exponential factor ( 50 values tested, see Additional file 1: Text S3). The infection risk for $s_{i}$ is determined by $\rho_{i}(hci)=\beta_{i}$, and a higher value means a greater extent of the transmission in the community.

#### Human flow intensity (HFI).

The infection risk caused by people entering and leaving a community was defined by hourly counts of overall inflow and outflow. The $\rho_{i}$ in the community $s_{i}$ is expressed as $\rho_{i}(hfi)=\sum_{t=0}^{T}\;P_{\to i}^{t}+P_{i\to }^{t}$, and T is the duration considered (e.g., T=48 hours under two-days human mobility patterns).

#### Case flow intensity (CFI).

Hourly counts of initial confirmed cases depicted the infection risk due to transmission events. At hour t−1, there are $C_{j}^{t-i}$ and $C_{i}^{t-1}$ initial confirmed cases in communities $s_{j}$ and $s_{i}$, respectively. In terms of the inter-community movement of initial confirmed cases, $P_{ji}^{t}$ people travel to the community $s_{i}$ from $s_{j}$ at hour t, of which the number of the initial confirmed cases is positively proportioned to the population flow, that is $C_{ji}^{t}=C_{j}^{t-1}\cdot \frac{P_{ji}^{t}}{P_{j}^{t-1}}$. A total of $\sum_{s_{j}\in M_{\to i}^{t}}C_{ji}^{t}$ enter and $\Sigma_{s_{k}\in M_{i\to }^{t}}C_{ik}^{t}$ leave the community $s_{i}$. The number of the initial confirmed cases at hour t is given by $C_{i}^{t}=\sum_{s_{j}\in M_{\to i}^{t}}C_{ji}^{t}+C_{i}^{t-1}-{\text{Σ}}_{s_{k}\in M_{i\to }^{t}}C_{ik}^{t}$, and the $\rho_{i}$ for the $s_{i}$ is expressed as $\rho_{i}(cfi)=\sum_{t=0}^{T}\;C_{i}^{t}$.

#### Case transmission intensity (CTI).

The infection risk due to transmission events was depicted by hourly counts of potential new infections caused by the initial confirmed cases within a community. At hour t, in terms of intra-community movement of $C_{i}^{t}$ initial confirmed cases within $s_{i}$, new infections increased with the infection rate given by $\lambda_{i}=\beta_{i}\cdot \frac{c_{i}^{t}}{p_{i}^{t}}$, where $\beta_{i}$ is the intra-community transmission rate derived from the logged POI-based diversity index. The number of new infections in the community $s_{i}$ at hour t is $I_{i}^{t}∼Binom\left(P_{i}^{t}-C_{i}^{t},\lambda_{i}\right)$ [41], and $\rho_{i}(cti)=\sum_{t=0}^{T}\;I_{i}^{t}$.

We denoted the day when the first case was reported for an outbreak as $d_{1}$. Subsequently, we examined hourly inter-community population flows representing mobility patterns during the initial two days (i.e., $d_{1}$ and $d_{2}$). For our analysis, we selected confirmed cases reported from day $d_{3}$ to $d_{4}$ as the initial cases, allowing for flexibility in the choice of initial case selection (see different selections of initial cases over time in Additional file 1: Table S7). In this context, the start hour, t=0, represented the first hour of day $d_{1}$, with the analysis covering a duration, T, of 48 hours. Furthermore, $C_{i}^{t=0}$ denoted the total number of confirmed cases reported in community $s_{1}$ from day $d_{3}$ to $d_{4}$

#### Susceptible-Exposed-Infectious-Removed (SEIR) epidemiological model.

The study employed a travel network SEIR modeling framework to simulate the spread of COVID-19 within city communities [42]. Simulation parameters and the commencement date were determined using the BEARmod framework (https://github.com/wpgp/BEARmod), with details provided in Additional file 1: Text S2, referencing existing studies. The model output, representing the daily cases in each community, was derived from a single simulation. Cumulative cases per community during the outbreak were computed. The community-level infection risk (SEIR-informed $\rho_{i}$) was established by averaging results from multiple simulations (e.g., 500). A comparison between the SEIR model’s disease transmission estimates and the actual COVID-19 outbreak spread is depicted in Additional file 1: Fig. S6. Additionally, the sensitivity of SEIR estimates to various values of R_0_ was assessed, as illustrated in Additional file 1: Fig. S7.

### Performance assessment of mobility-based spatial sampling

**Fig. 2.**
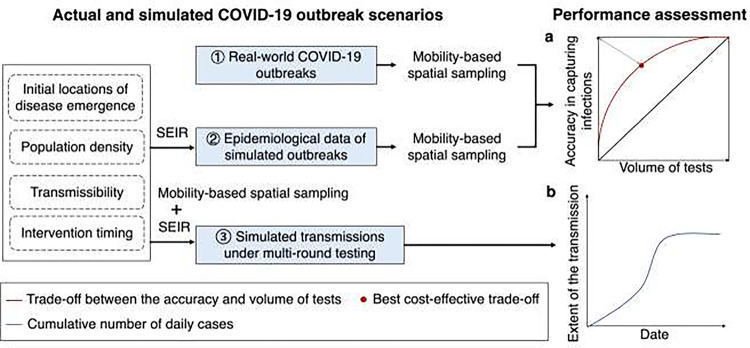
Framework of assessing the performance of mobility-based spatial sampling approaches to detect emerging infections at the community level. Based on actual COVID-19 outbreaks and simulated outbreaks using an epidemiological model (SEIR) under the different transmissibility, intervention, and population density scenarios, trade-offs between the volume of tests and the detection of infections throughout an outbreak were employed to estimate the performance of sampling approaches, where the red curve and black diagonal represent the performance of the mobility-based sampling and simple random sampling, respectively. The red dot on the red curve with the least geometric distance to the upper left corner was considered the best cost-effective trade-off. Additionally, spatial sampling was incorporated into SEIR to simulate the disease transmission under multiple rounds of mass testing, where the cumulative number of estimated cases depicted the extent of the transmission within a city. Less cases under an outbreak using a sampling approach indicated a more significant effect on interrupting the spread of the disease.

The study comprehensively assessed the effectiveness of mobility-based spatial sampling in three distinct scenarios. First, the evaluation focused on the practical application of mobility-based sampling to improve community-level testing for detecting infections during real-world COVID-19 outbreaks. The assessment involved measuring the accuracy of infection detection at the community level and the volume of tests conducted. The trade-off between these factors was analyzed at different sampling sizes, aiming for an optimal balance. To assess the accuracy of infection detection in space and quantity, the study measured the proportion of affected communities or cases that were successfully sampled over the total number of affected communities or cases throughout an outbreak. The volume of tests was evaluated by calculating the ratio of sampled communities or populations over the total number of communities or people. In an ideal scenario, a perfect sampling approach would yield a point as close as possible to the upper left corner in Fig. 2a. This would mean that all infections could be precisely detected using a sample size that is equivalent to the number of cases or affected communities. Practically, the study used the point with the least geometric distance to the upper left corner (the red point) as the best cost-effective trade-off. This point represented the most balanced compromise between test accuracy and volume. The assessment revealed that, aside from the red point, there were situations where increasing accuracy came at the cost of conducting more tests or where reducing accuracy required fewer tests. Additionally, the average performance of each sampling method was quantified using the area under the red curve, providing an overall measure of its effectiveness.

Secondly, the study explored the applicability of mobility-based sampling in simulated epidemics, considering various outbreak and data scenarios that encompassed different aspects such as initial disease emergence locations, transmissibility, population density, and intervention timing. The performance of each sampling approach was assessed in each scenario, gauged by the area under the red curve.

Lastly, spatial sampling was integrated into the SEIR model to simulate disease transmission under multi-round testing, providing an evaluation of the sampling approach’s effectiveness in mitigating the spread of the epidemic. The extent of simulated transmission within a city was represented by the cumulative number of cases, with fewer cumulative cases indicating a more substantial impact of the sampling on interrupting disease spread (Fig. 2b).

### Multi-round testing with mobility-based spatial sampling

To evaluate how mobility-based sampling can enhance the implementation of multi-round testing in detecting infections, spatial sampling was integrated into an SEIR model (Additional file1: Text S6). This integration facilitated the simulation of disease transmission under multiple rounds of testing. The cumulative number of cases was employed to quantify the extent of the simulated transmission within a city. A reduction in the cumulative cases throughout an outbreak signified a more pronounced effect of the sampling approach in augmenting the effectiveness of mass testing for controlling the epidemic’s spread.

The simulation involved four approaches combined with multiple rounds of large-scale testing. The baseline approach allocated daily testing resources equally to all communities within a city. In contrast, the SRS, CFI, and CTI approaches sampled a specified number of communities per day and allocated more resources to the sampled communities than those that were not sampled. While each community had the same probability of being sampled using SRS, communities with higher infection risk had a greater probability of being sampled using CFI or CTI.

Across all outbreak scenarios, the SEIR model’s simulation started on the same day as the real-world outbreak in Guangzhou and Beijing. The initial stage of the epidemic was simulated using SEIR for the first four days following the outbreak. Infection risks derived from CFI and CTI were calculated based on the initial cases and the human mobility patterns of the first two days within the city.

Mass testing was assumed to commence on the fifth day of the outbreak (or until the twelfth day in scenarios with interventions delayed by one week) and last for 12 days. In the SRS/CFI/CTI approaches, 1/12 of all communities were sampled each day, and multiple rounds of testing could be conducted in a community over the 12 days due to the randomness of sampling. Importantly, the total testing resources for a city remained equivalent across the different approaches, ensuring a fair comparison.

## Results

### Enhancing infection detection efficiency in real-world COVID-19 outbreaks

**Fig. 3.**
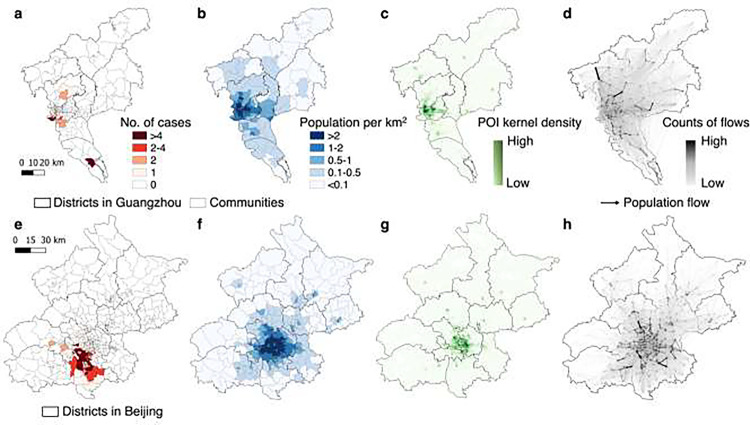
Overview of the data context of real-world COVID-19 outbreaks in Guangzhou and Beijing. **a** and **e**, Geospatial distributions of cases at the community level during the importation-related outbreaks. **b** and **f**, Geospatial distributions of community-level population density, which were classified into five levels. **c** and **g**, Geospatial patterns of point-of-interest (POI) kernel density. **d** and **h**, Human mobility patterns across communities within a city before travel restrictions are implemented. The directed lines depict inter-community origin-destination travel networks on 21–22 May 2021 in Guangzhou and 11–12 June 2020 in Beijing, respectively. The width and color of an edge represent the volume of an inter-community flow. In each panel, a darker color indicates a higher level of interest.

Fig. 3 provides a comparative analysis of COVID-19 transmission scenarios and outbreak data in Guangzhou and Beijing, illustrating the distinct geospatial patterns observed in the two cities during the outbreaks. In the case of Beijing, the affected communities with reported COVID-19 cases were spatially clustered, covering a higher density of communities than observed in Guangzhou (Figs. 3a and 3e). Both cities exhibited similar geospatial distributions of population and POI density, with urban areas being prominent concentration points (Figs. 3b and 3f). Notably, several communities across different districts displayed concentrated POI clusters, denoting high activity levels (Figs. 3c and 3g). However, the mobility patterns between communities in Beijing and Guangzhou differed significantly (Figs. 3d and 3h). In Guangzhou, individuals exhibited extensive movement between communities, even those located far apart and in different districts. On average, individuals within a specific community visited approximately 96.6% of all communities within Guangzhou in a single day (Additional file 1: Fig. S3a). This proportion was calculated by determining the cumulative number of distinct communities that individuals from a particular community visited within a single day. Conversely, inter-community movements in Beijing were predominantly intra-district, primarily occurring in the south and east. Individuals from one community visited only about 59.4% of the communities, reflecting a more localized pattern of movement.

**Fig. 4.**
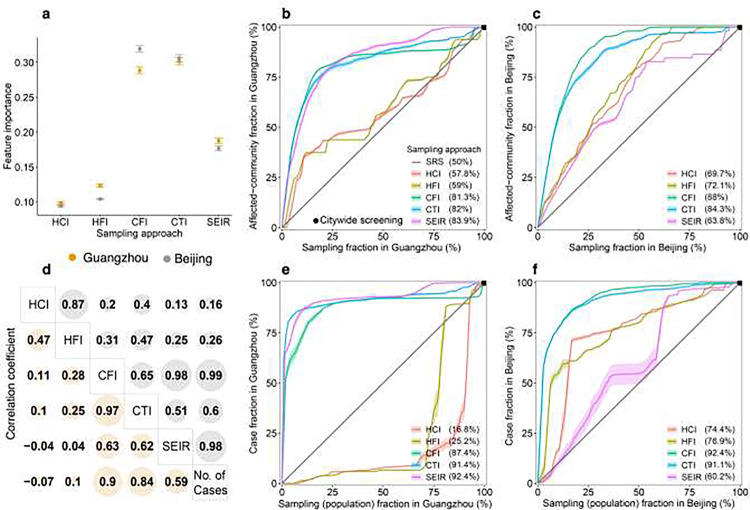
Performance of mobility-based spatial sampling approaches in detecting COVID-19 affected communities and cases at varying sample sizes. Four mobility-based spatial sampling approaches (HCI - human contact intensity; HFI - human flow intensity; CFI - case flow intensity; CTI - case transmission intensity) and an epidemiological model (SEIR) were evaluated. **a**. The relative importance of infection risk ($\rho_{i}$) in distinguishing communities with COVID-19 cases from those without, determined by a random forest built-in feature importance measure. Error bars indicate 95% confidence intervals. **d.** Pearson correlation coefficients between infection risk estimated from each sampling method and the number of confirmed cases during the outbreaks. For panels **b-c** and **e-f**, communities with high infection risk were sampled by ranking community-level 𝜌_𝑖_ from high to low, excluding the simple random sampling (SRS) method. The x-axis in **b** and **c** represents the proportion of sampled communities over the total number of communities in Guangzhou and Beijing, respectively. In **e** and **f**, the x-axis denotes the fraction of sampled populations among the total populations. The y-axis in **b** and **c** represents the proportion of affected communities sampled over the total communities with COVID-19 cases in Guangzhou and Beijing. In **e** and **f**, the y-axis displays the proportion of cases detected by different sampling approaches among the total cases. The percentage in the legend indicates the area under each curve, reflecting the average performance of each sampling approach with different sample sizes. The black dot at the upper right corner of each panel represents citywide screening for the entire population, assuming the test can detect all infected people in the city. Shaded regions denote 95% confidence intervals.

In the identification of communities affected by COVID-19 during outbreaks in Guangzhou and Beijing, the CFI and CTI approaches exhibited superior performance over the HCI and HFI methods, when COVID-19 testing was conducted in communities sampled by these approaches. In comparison to the travel network-based SEIR model, CFI and CTI demonstrated enhanced accuracy, especially when fewer communities were sampled. The infection risk (𝜌_𝑖_) estimated by CFI and CTI played a pivotal role in effectively distinguishing affected communities, surpassing the performance of the SEIR model, HCI, and HFI (Fig. 4a). Optimal cost-effective trade-offs for CFI were identified when sampling 17.9% and 21.1% of communities in Guangzhou and Beijing, respectively. These percentages allowed for the detection of 78.5% and 84.1% of affected communities in the respective cities (Figs. 4b and 4c).

Moreover, CFI and CTI markedly enhanced the efficiency of case detection. Infection risks estimated by CFI, CTI, and SEIR exhibited statistically significant correlations with the number of confirmed cases during the outbreaks (Fig. 4d). For optimal cost-effective trade-offs, utilizing CFI and CTI to sample only 15.7% and 7.2% of the population in Beijing and Guangzhou, respectively, enabled the identification of 85.1% (95% CI: 84.9–85.3) and 85.5% (85–85.9) of reported cases during the outbreaks (Figs. 4e and 4f). Mobility-based spatial sampling, as facilitated by CFI and CTI, significantly reduced the sample size and testing volume compared to citywide screening and SRS, while maintaining detection accuracy. For example, in Guangzhou, CFI and CTI identified, on average, 37.4% and 41.4% more cases than SRS, and in Beijing, they detected, on average, 42.4% and 41.1% more cases than SRS.

The study conducted a comparison between deterministic and Poisson methods across various sampling approaches (Additional file 1: Fig. S1). When employing equivalent approaches and sample sizes, the average accuracy of Poisson-based CFI and CTI methods was 6.6% and 4.1% lower, respectively, compared to the deterministic method. Moreover, the SEIR model performed better in detecting affected communities and cases in Guangzhou compared to Beijing (Additional file 1: Table S6), likely due to the challenge in estimating the wider spread of the disease in Beijing, given its highly heterogeneous mobility network.

### Effectiveness of spatial sampling in simulated outbreak and data scenarios

**Fig. 5.**
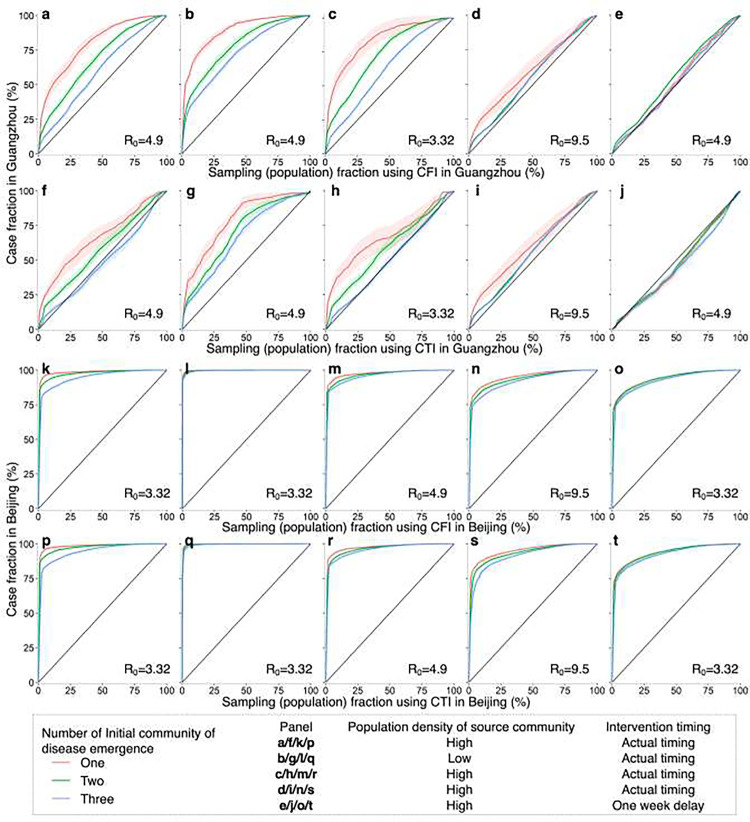
Performance of mobility-based spatial sampling in simulated outbreaks under various scenarios utilizing a travel network-based epidemiological model. The outbreaks were simulated with initiation in one, two, or three communities selected randomly based on the probability weight of population density or inverse population density. Different basic reproduction numbers (R_0_) were considered for the original SARS-CoV-2, Delta, and Omicron variants, along with variations in the timing of interventions. The assessment focused on two optimized mobility-based spatial sampling approaches, namely CFI (case flow intensity) and CTI (case transmission intensity). The x-axis represents the fraction of sampled populations among the total populations in Guangzhou and Beijing using CFI and CTI, respectively. The y-axis presents the proportion of cases detected by different sampling approaches in Guangzhou and Beijing, respectively. The diagonal line in each panel symbolizes the performance of simple random sampling, while the shaded regions indicate the 95% confidence intervals.

The performance of CFI and CTI was further assessed through simulations of outbreaks in diverse settings, incorporating variations in initial disease emergence locations, transmissibility, population density, and mobility-mediated spread within a city over time. In simulated outbreaks, both approaches consistently outperformed SRS in terms of spatial coverage and quantity of detected infections. Notably, their effectiveness was more pronounced under conditions involving fewer initially affected communities, low population-density communities as the outbreak origin, smaller R_0_, and prompt implementation of public health interventions (Fig. 5, Additional file 1: Table S2). The accuracy of CFI and CTI in identifying affected communities or cases diminished as the geographic extent of epidemic transmissions across communities increased (Additional file 1: Fig. S2). For instance, when R_0_ equaled 9.5, representing the Omicron variant [43], or non-pharmaceutical interventions experienced a one-week delay, the use of CFI and CTI did not confer a significant advantage in Guangzhou, as the disease may have already disseminated to most communities (87.7%–92.7%) in the city (Figs. 5d–e and 5i–j).

Furthermore, in simulated outbreaks in Guangzhou, CFI exhibited a higher average accuracy than CTI, whereas their accuracy was nearly identical in Beijing. Simulated outbreaks affected a larger proportion of communities in Guangzhou than in Beijing under the same initial settings. CFI and CTI performed better in Beijing, improving the efficiency of detecting emerging infections. However, their performance was higher in Guangzhou than in Beijing when the inter-community mobility characteristics were exchanged between the two cities (Additional file 1: Fig. S3).

### Optimizing infection detection through multi-round testing with spatial sampling

**Fig. 6.**
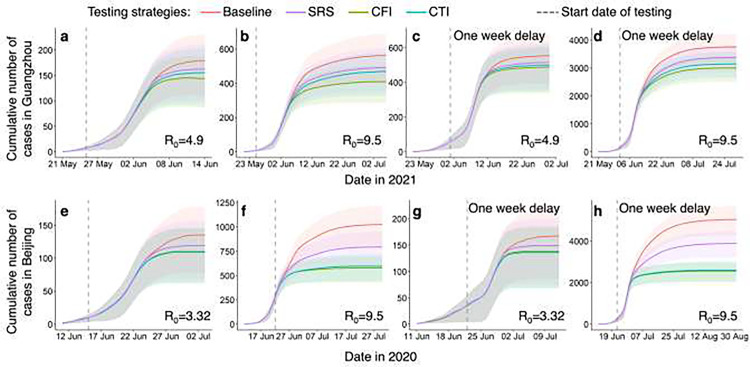
Impact of spatial sampling on multi-round testing for detecting infections to contain transmission. The simulations for Guangzhou and Beijing scenarios are presented in panels **a-d** and **e-h**, respectively. Multiple rounds of testing for detecting infections were implemented using spatial sampling and incorporated into the travel network-based epidemiological model (Additional file 1: Text S6). The epidemiological model simulated the epidemic transmission, measured by the daily cumulative cases, under different sampling approaches and outbreak scenarios. The baseline approach of multi-round testing involved the equal allocation of daily testing resources to all communities within a city. However, simple random sampling (SRS), case flow intensity (CFI), and case transmission intensity (CTI) sampled a given number of communities per day and allocated more resources to sampled communities than unsampled areas. Spatial multiple rounds of testing were executed when a community could be sampled several times. The outbreaks were tested under different settings, including various basic reproduction numbers (R_0_) of the original SARS-CoV-2, Delta, and Omicron variants, and the timing of testing conduction. Detection testing started on the fifth day of an outbreak for panels **a-b** and **e-f**, while it began on the twelfth day of the outbreak for panels **c-d** and **g-h**. The shaded regions represent the interquartile ranges of the cumulative number of daily cases in the simulated outbreaks.

To assess the effectiveness of the CFI and CTI approaches in detecting and isolating infected individuals during outbreaks caused by highly contagious pathogens, we investigated the integration of spatial sampling with multiple rounds of detection testing. Our results indicate that, compared to a baseline approach where daily testing resources were equally distributed across all communities in a city, multi-round testing with CFI or CTI sampling could lead to earlier detection and containment of transmission under various outbreak scenarios (Fig. 6). These mobility-based approaches optimally allocated limited testing resources to high-risk communities sampled each day. Specifically, under outbreaks with a higher R_0_, multi-round screening integrated with CFI/CTI demonstrated superior performance in detecting infections to contain transmission (Additional file 1: Table S3). For example, compared to the baseline approach, CFI could reduce cases in Guangzhou and Beijing by 27.8% (26.1–29.6) and 43.8% (42.6–45.1), respectively, with an R_0_ equal to 9.5. However, the reduction in infections achieved by CFI was 19.3% (18–20.7) under an R_0_ of 4.9 in Guangzhou and 18.7% (17.7–19.7) under an R_0_ of 3.32 in Beijing. Notably, the average effect of CFI for multi-round testing was superior to that of CTI in Guangzhou, while the effects of both were almost identical for simulated outbreaks in Beijing. Furthermore, a delayed testing conduction would result in a significant increase in community transmission. For instance, if testing for detecting infections were delayed by one week in Beijing, the total number of cases would be four times higher than that observed with the actual timing of testing.

## Discussion

Early identification of cases is a critical component in controlling outbreaks and mitigating the spread of EIDs. During the COVID-19 pandemic, a range of intervention measures, including mass testing, were implemented to enhance case detection and contain the transmission of SARS-CoV-2 and its variants [32, 44]. Despite the widespread use of mobile phone-based mobility data to understand the spread of infectious diseases and the impact of interventions [45–47], its potential for optimizing the identification of emerging infections requiring testing has been underexplored. This study demonstrates that leveraging human mobility and POI data through mobility-based spatial sampling can markedly reduce the number of individuals screened while enhancing the efficiency of detecting emerging infections at the community level, all while maintaining a high accuracy in infection identification.

The findings underscore the potential enhancement in the performance of community-level testing through thoughtful consideration of initial confirmed case locations and mobility patterns within and between communities. Both HCI and HFI tend to sample areas with high human activity, which may not necessarily align with the areas where cases are present due to timely public health interventions. This mismatch can lead to resource inefficiencies and hinder testing efficacy, as observed in the Guangzhou outbreak (Fig. 3). Consequently, spatial sampling approaches that integrate human mobility data with epidemiological insights in the early stages of an outbreak can significantly enhance infection detection efficiency. In this regard, the CFI and CTI approaches, which consider both inter- and intra-community movements of initially affected populations in communities reporting cases, demonstrated superior performance compared to other geospatial sampling methods. For instance, using CFI and CTI enabled the detection of over 85% of infections by sampling less than 16% of the populations during COVID-19 outbreaks in Guangzhou and Beijing (Fig. 4). While sampling 16% of the populations in the two cities equates to testing individuals numbering in the millions, it significantly reduces resource waste by markedly reducing the volume of tests compared to citywide screening. SEIR models, while contributing to improved efficiency in case identification by estimating transmission risks, are inherently complex and reliant on various epidemiological assumptions and parameters. In contrast, CFI and CTI, with fewer epidemiological assumptions and parameters, offer stability and ease of use in various scenarios, especially during the early stages of a pandemic when rapid response decision-making is critical.

The effectiveness of CFI and CTI varied across different simulated transmission scenarios, outbreak data, and parameter scenarios. Both CFI and CTI demonstrated significant performance in situations involving the transmission across a few communities within a city. Notably, CFI appeared to be more stable than CTI, especially in the context of simulated outbreaks in Guangzhou (Fig. 5). The CTI approach introduced additional uncertainties due to assumptions related to parameters for estimating transmission events caused by the movement of cases within communities. Additionally, CTI and SEIR estimated spatial infection risk in different ways, leading to inconsistencies when applying CTI to outbreaks simulated by the SEIR model, particularly when transmission events occurred in many communities. Balancing the complexity of indicators with practicality is crucial, and the findings suggest that excessively intricate models may not necessarily provide linear improvements in depicting infectious disease transmission dynamics.

However, the efficacy of both CFI and CTI diminished as the geographic extent of epidemic transmissions across communities increased owing to outbreak scenarios covering densely populated areas, high disease transmissibility or delayed intervention (Fig. 5). In these scenarios, the disease may have spread to most communities within a city and had entered a phase of widespread community transmission (Additional file1: Fig. S2). Mobility-based sampling had limited effectiveness in detecting infections at the community level. Stringent measures such as citywide screening and lockdowns were crucial to interrupt community transmission. Moreover, the effectiveness of CFI and CTI decreased with the increase of mobility for high-impact communities (Additional file1: Fig. S4). Imposing mobility restrictions across communities became imperative, particularly in cities where population flows encompassed a majority of communities. For instance, an outbreak in a single community in Guangzhou could affect numerous communities, even with a small R_0_, as individuals from that community visited most of Guangzhou’s communities in a single day. Nonetheless, during the early stages of an outbreak, CFI or CTI could improve the effectiveness of mass testing in suppressing disease spread by optimizing the allocation of testing resources across various geographic ranges and temporal frequencies, even under conditions of high disease transmissibility or delayed interventions (Fig. 6).

In summary, our study highlights the potential of implementing CFI and CTI to enhance infection detection efficiency, especially in the early stages of infectious diseases when the epidemic is localized. To begin, early adoption of CFI and CTI facilitates prompt detection of infections to support for the containment of subsequent epidemic propagation. Furthermore, in situations where outbreaks occur within densely populated regions with high levels of inter-community population mobility, the effectiveness of CFI and CTI may be attenuated, underscoring the necessity for immediate responses and the enforcement of rigorous control measures. Additionally, CFI and CTI can effectively identify high-risk communities, thereby enabling targeted, multi-round, and high-frequency mass testing to contain emerging outbreaks of infectious diseases. It indicates that implementing CFI or CTI as part of comprehensive strategies, such as city-wide test-trace-isolate approaches, promptly is vital for highly transmissible diseases.

This study has several notable limitations. Firstly, the study faced constraints in accessing only a short period of mobility data before travel restrictions were imposed in Guangzhou and Beijing due to data availability. This is a common challenge in early-stage epidemic response, where real-time and limited data are frequently employed for decision-making. The inclusion of longer time series of population movements could enhance the accuracy of mobility-based sampling methods. While our study provides valuable insights into early risk assessment and testing optimization, future research should explore the performance of sampling methods as an outbreak progresses into later stages. Secondly, direct verification of the reliability of the widely used and validated POI and mobility data was challenging. Nonetheless, the reliability of the proposed methods was improved through various sensitivity analyses conducted on data, models, and parameters. Thirdly, this study did not account for any interventions applied in conjunction with testing or constraints assumed to apply to identified cases. These factors could potentially influence the number of infections sampled and identified. Nevertheless, it is important to note that the sampling approaches employed in this study do not directly impact the accuracy of testing. Fourthly, this study relied on data from COVID-19 outbreaks in Guangzhou and Beijing, as well as simulated epidemics. To comprehensively validate and extend the effectiveness of the proposed approaches, it’s recognized that a more extensive dataset encompassing various infectious diseases may be necessary. Lastly, metapopulation-based models were employed to represent population aggregates at the community level in a city. This was due to the unavailability of individual-level trajectory data, and limitations in characterizing higher-order interactions between individuals at a large spatial scale [48–50]. To account for multiple interaction patterns affecting epidemic transmission, the models considered the randomness and heterogeneity of the transmission process for different mobility scenarios and epidemiological parameter combinations (Additional file1: Figs. S3-S5).

## Conclusion

The study demonstrates the potential of leveraging information on human movement and contact patterns to enhance the effectiveness of spatial sampling. The proposed mobility-based spatial sampling approach offers a substantial improvement in the efficiency of community-level testing for detecting emerging infections. It achieves this by reducing the number of individuals screened while maintaining a high accuracy rate in identifying infections. Furthermore, this approach can pinpoint high-risk communities, facilitating targeted, multi-round, and high-frequency mass testing in containing disease transmission. By utilizing mobility-based spatial sampling, a cost-effective solution is provided for the optimal allocation of testing resources and early surveillance of intra-city transmission. This approach proves valuable in mitigating emerging outbreaks of infectious diseases in diverse settings.

## Data Availability

All source code and processed data are available and accessible at GitHub repository (https://github.com/zhangdie12138/COVID-19Sampling).

## Abbreviations

COVID-19: Coronavirus disease 2019
SARS-CoV-2: Severe acute respiratory syndrome coronavirus 2
POI: Point-of-interest
SRS: Simple random sampling
HCI: Human contact intensity
HFI: Human flow intensity
CFI: Case flow intensity
CTI: Case transmission intensity
SEIR: Susceptible-Exposed-Infectious-Removed
CI: Confidence interval
